# Surgical inflammation: a pathophysiological rainbow

**DOI:** 10.1186/1479-5876-7-19

**Published:** 2009-03-23

**Authors:** Jose-Ignacio Arias, María-Angeles Aller, Jaime Arias

**Affiliations:** 1 General Surgery Unit, Monte Naranco Hospital, Oviedo, Asturias, Spain; 2 Surgery I Department, School of Medicine, Complutense University of Madrid, Madrid, Spain

## Abstract

Tetrapyrrole molecules are distributed in virtually all living organisms on Earth. In mammals, tetrapyrrole end products are closely linked to oxygen metabolism. Since increasingly complex trophic functional systems for using oxygen are considered in the post-traumatic inflammatory response, it can be suggested that tetrapyrrole molecules and, particularly their derived pigments, play a key role in modulating inflammation.

In this way, the diverse colorfulness that the inflammatory response triggers during its evolution would reflect the major pathophysiological importance of these pigments in each one of its phases. Therefore, the need of exploiting this color resource could be considered for both the diagnosis and treatment of the inflammation.

## Background

The inflammatory response related to surgery (elective or anesthetized injury) and to trauma (accidental or unanesthetized injury) could be considered a surgical inflammation [1]. The surgical inflammation, as an inflammatory process, could be viewed as composed of a series of overlapping successive phases [2]. That is why it is common that each researcher chooses for his study a specific aspect of this complex response. At the same time, the interrelation of the knowledge that is successively obtained allows for better understanding the pathophysiological mechanisms of the surgical inflammation. It also allows for suggesting new possible meanings of this inflammatory response.

Color is a quality of the surgical inflammation that has always been observed. The color in inflammation is one of the components by which the classical description of inflammation accounts for the visual changes observed. Based on visual observation, the ancients characterized inflammation by four cardinal signs, namely redness, swelling, heat and pain [3].

It could be considered that the color of the injured tissue is changeable because both the traumatic injury (contusion and/or wound) and the inflammatory response related to this aggression are evolutive. The post-traumatic acute inflammatory response has especially been described as a succession of three functional phases with increasingly complex trophic functional systems for using oxygen [2,4]. It is considered that also the state of wound oxygenation is a key determinant of healing outcomes [5]. And, interestingly enough, it could be imagined that an array of colors is displayed through this evolution. Therefore, it could be considered that tetrapyrrole molecules, such as heme, in addition to contributing a large variety of colors to the tissues, are employed through the evolutive process of acute inflammation. The great variability of tetrapyrrole end-products, diversified both in plant and animal life during the evolution of eukaryotic cells could mean an adaption to the metabolic and biochemical changes imposed by the development in different environments, from an unbreathable atmosphere to an environment fully enriched by oxygen [2].

### Tissue injury and inflammation

#### - Tissue injury

In mechanical trauma, it is considered that the inflammatory response is induced by tissue injury [1,2]. However, its special initial superimposition suggests that a continuous pathophysiological mechanism is established.

Tissue injury due to mechanical energy can produce a contusion (bruise), that is, damage without tissue breakage or damage with tissue breakage. In this last case, if the tissue is soft, the lesion is called a wound and if the tissue is hard, the lesion is called a fracture [6].

**Figure 1:**
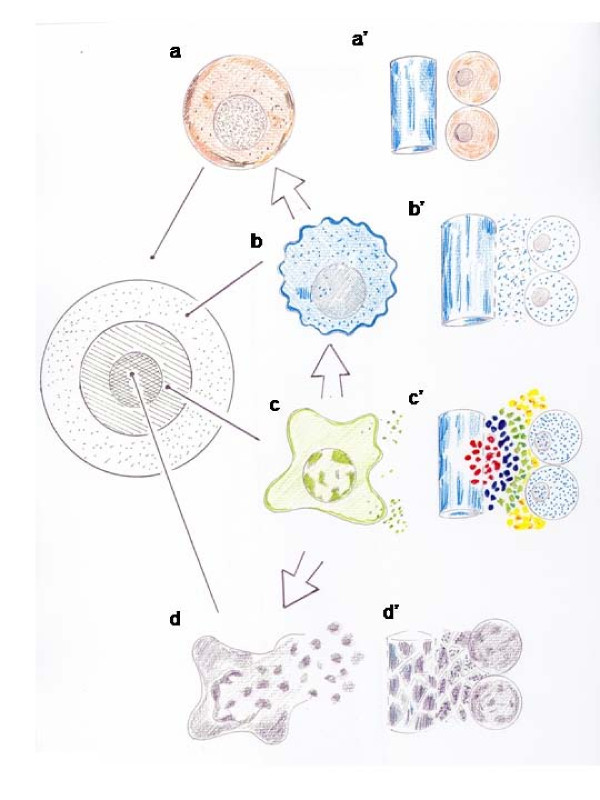
**Degrees of severity in the contusions**. Injury without breakage produced by blunt etiological agents and are made up of concentric areas of different degrees of severity. From the cellular point of view, the first-degree contusion is a reversible injury. The alteration consists in small plasma bleb formation. In the second-degree contusion, a fusion of the blebs is produced and the plasma membrane permeability increases. In the third-degree contusion, cell death is produced by necrosis. At the same time, contusions can be superficial or deep. From the tissue point of view, edema is produced in the first-degree contusion; ecchymosis would be associated with edema in the second-degree contusion; an infarction would be produced in the third-degree contusion. Ecchymosis means that the red blood cells are the first blood cells to infiltrate the interstitial space in post-traumatic inflammation. Ecchymosis, also called a contusion or a bruise, due to its blue color, from the Latin word *cardinus *(bluish) explains its purple color.

The contusion, based on its severity, could be classified in three degrees: first degree, characterized by the temporary loss of function. Although it could be associated with edema, the alterations are reversible, and therefore, full recovery is possible. Second degree would occur with ecchymosis, namely with tissue infiltration by red blood cells. The evolution would be ambivalent since cellular and tissue alterations can be reversed or worsened, causing cell death. Thus, the oxygen plays a key role in the evolution of the second degree contusions since extreme near anoxic environment is not compatible with tissue repair [5]. And lastly, the third degree is an irreversible lesion since the injury causes cell death by necrosis and the tissue suffers from infarction [6] (Figure 1).

**Figure 2:**
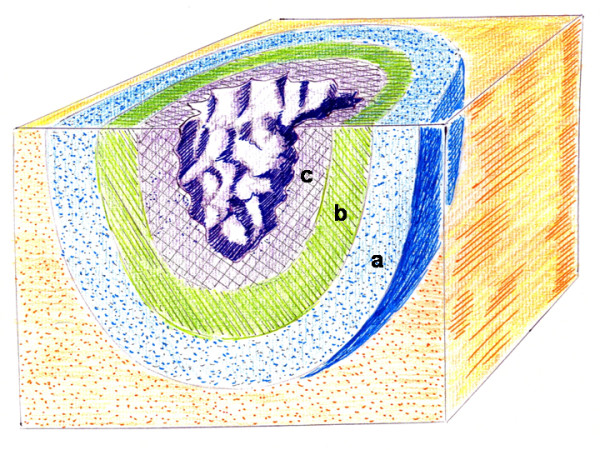
**Schematic representation of a wound**. Injury without breakage in the soft tissue can be superficial or deep. The contusive wounds induce a first, second and third-degree contusion in the tissues, as the figure shows. The evolution of the second-degree bruised area, whether reversible or irreversible, will determine the evolution of the wound since it can increase the necrosis area. The superficial injury with breakage has external hemorrhaging and the deep injury without breakage has contusions of internal tissue or intraparenchymatous hemorrhaging. a: first-degree contusion; b: second-degree contusion; c: third-degree contusion

Cellular and tissue lesion is irreversible in the wound and fracture since necrosis is produced. [6]. Particularly, the wound enters the tissue suffering from a first, second or third-degree overlapped contusion areas, as the figure 2 shows. In the third-degree contusion area, anoxia avoids the wound repair. The evolution of the second-degree bruised area, whether reversible or irreversible, will determine the evolution of the wound since it can increase the necrosis area. Hypoxia in this area could be mild or modest. At last, in the first-degree contusion area, that is the most peripheral area around the wound, the inexistence of hypoxia avoids the complications development and, therefore it does not affect the tissue viability.(Figure 2).

Until recently, necrosis has often been viewed as an accidental and uncontrolled cell death process. Nevertheless, growing evidence supports the idea that necrotic cell death may also be programmed [7]. Cellular signaling events have been identified to initiate necrotic destruction that could be blocked by inhibiting discrete cellular processes [8]. The most relevant mechanisms culminating in cell necrosis correspond to mitochondrial dysfunction and ATP depletion; loss of intracellular ion homeostasis with osmotic swelling and oxidative stress; activation of degrative hydrolases, including proteases, phosphorylases, and endonucleases; and degradation of cytoskeletal proteins with disruption of cytoskeletal integrity [9]. Surprisingly enough, this list of mechanisms also corresponds to those that occur in the acute inflammatory post-traumatic response [2,4]. It seems, that in response to injury, cells can develop a mechanism that would play a defensive role (inflammation) and that could favor reversing the alterations until their inadequate expression would make them harmful (necrotic). Hence, at a specific moment in time, the pathophysiological mechanisms (cellular response to injury) become pathogenic mechanisms (producers of cell death) [4].

#### - Tissue Inflammation

We have proposed that the acute inflammatory response to injury by mechanical energy, regardless of whether it is local or systemic, is based on the successive pathologic functional predominance of the nervous, immune and endocrine systems. This hypothesis implies that the final and prevalent pathologic functions of these systems may represent the consecutive phases of the response to stress developed by the body, all of which may have a trophic meaning for the injured tissue [4,10].

Perhaps the leading role in this response is played by the relation between the blood and the interstitial space. This assumption is based on the fact that the different blood components escape the intravascular space one by one in order to occupy the interstitial space, where they play the main role in the successive phases of the inflammatory response. Therefore, the endothelium plays a bidirectional mediating role between blood flow and the interstitial space, which is where inflammation mainly takes place [2,4].

Since the phases of the inflammatory response go from ischemia to the development of an oxidative metabolism, the successive pathophysiological mechanisms that develop in the interstitium of tissues when they undergo inflammation are considered increasingly complex trophic functional systems for using oxygen [2,4,10].

#### - Phases of the Inflammatory Response

**Figure 3:**
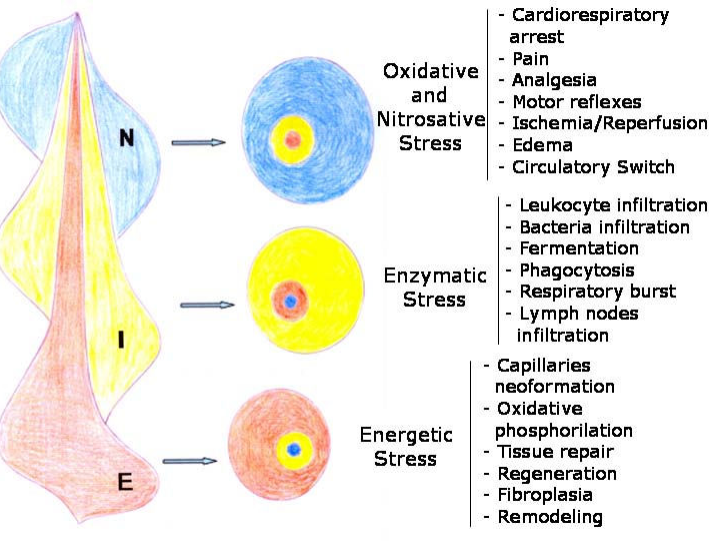
**Phases of the post-traumatic inflammatory response**. The post-traumatic inflammatory response is considered to be made up of three overlapping phases with increasingly complex trophic functional systems for using oxygen. During the first or nervous phase, oxidative and nitrosative stress are produced. In the second or immune phase, enzymatic stress is produced and in the third or endocrine phase, oxidative phosphorylation is reached and therefore, energetic stress is produced. N: Nervous phase with oxidative stress and edema which progressively subsides(blue). I: Immune phase with enzymatic stress and its subsequent neutralization(yellow). E: Endocrine phase with its initial tissue de-structuring and subsequent tissue repair through regeneration and/or fibroplasia.(red).

It could be considered that the acute post-traumatic inflammatory response is made up of three overlapping phases, whether local or systemic (Figure 3).

The first or immediate phase has been referred to as the nervous phase, because the sensory (pain and analgesia) and motor alterations (contraction and relaxation) respond to the injury [2,4]. This early pathological activity, in essence, could reflect the predisposition of the body's nociceptor nervous pathways to first suffer depolarization with microglia activation and neuropeptide production. Furthermore, this nervous response coexists almost completely with the tissue injury evolution and, therefore, conditions it.

The nervous or immediate functional system presents ischemia-revascularization and edema, which favor nutrition by diffusion through the injured tissue. In reality, the tissues suffer ischemia-reoxygenation, that is, they begin using oxygen after a more or less long period of ischemia. It is likely that the magnitude of wound hypoxia is not uniformly distributed throughout the affected tissue, especially in large wounds [5]. This trophic mechanism has a low energy requirement that does not require oxygen (ischemia) or in which the oxygen is not correctly used, with the subsequent excessive production of reactive oxygen and nitrogen species (ROS/RNS) (reperfusion). In this phase, while the progression of the interstitial edema increases in the space between the epithelial cells and the capillaries, the lymphatic circulation is simultaneously activated (circulatory switch). Thus, the injured tissues adopt an ischemic phenotype (hypoxia) [4] (Figure 3).

In the following immune or intermediate phase of the inflammatory response, the tissues and organs which have suffered ischemia-reperfusion, are infiltrated by inflammatory cells and, sometimes, by bacteria. Interstitial inflammation is favored by the concurrent activation of hemostasis and complement cascades. In the tissues and organs which suffer oxidative stress, symbiosis of the inflammatory cells and bacteria for extracellular digestion by enzyme release (fermentation) and by intracellular digestion (phagocytosis) could be associated with a hypothetical trophic capacity. Improper use of oxygen persists in this immune phase and is also associated with enzymatic stress. Furthermore, lymphatic circulation plays a major role and macrophages and dendritic cells migrate to lymph nodes where they activate lymphocytes [2,4,11] (Figure 3).

It is considered that angiogenesis characterizes the last or endocrine phase of the inflammatory response, so nutrition mediated by the blood capillaries is established [2,4,5]. However, the angiogenic process becomes active early and excessive proliferation of endothelial cells takes place which, in turn, develops a great density of endothelial sprouts. Through this initial and excessive proliferation, the endothelial cells could successively perform antioxidant and anti-enzymatic functions. These functions would favor the evolution of the inflammatory response towards tissue repair through specialized capillary development. If so, it would be in this last phase of the inflammatory response when the process of angiogenesis would be responsible for tissue nutrition through capillaries. Oxygen got its name from "Principe Oxygen" which means the acidifying principle."Oxy" is from Greek and means sharp or acid; "gen" is also from Greek and means the origin of. Taken together, oxygen means "the origin of acid" [5]. Oxygen and oxidative metabolism are an excellent combination through which cells can obtain an abundant energy supply (energetic stress) for tissue repair by epithelial regeneration or wound healing [2,4,5,10,11] (Figure 3).

### The color of the inflammatory phases

The colors of inflammation can be represented in three groups:

#### - Cold colors

The tissue color that is initially associated with mechanical injury is white. When mechanical energy acts on the tissue, especially if this occurs through a blunt etiological agent, an abrupt crushing is produced that takes the blood out of the tissue. The bloodless tissue is white, a color that brings together the entire light spectrum, but if it continues to be crushed, it becomes ominous since it can signal sphacelation. Thus, in a third-degree contusion, the tissue suffers a crush injury with vasospasm, endothelial damage and thrombosis [12] (Figure 1).

Decreased transcutaneous oxygen tension, reduced arterial hemoglobin saturation and increased transcutaneous carbon dioxide tension revealed a reduction in blood flow and poor tissue perfusion as the earliest warning signs of shock and death [13]. Then, a shift to anaerobic metabolism is provided through the metabolic adaptation to hypoxia. Again the paleness, in this case generalized, implies a poor prognosis.

Blood loss remains a leading cause of traumatic death [14]. Control of bleeding and correction of intravascular volume are the hallmarks of conventional resuscitation after massive blood loss [14]. After cardiopulmonary resuscitation of trauma patients with cardiac arrest, the survival rates are only 0% to 5% [15,16]. Cardiac resuscitation (chest compression without ventilation) by bystanders is the preferable approach for resuscitation [17]. In blunt and/or penetrating trauma patients efforts should be withheld in case there is evidence of a significant time lapse since pulselessness, including lividity, *rigor mortis *and decomposition [18].

Early care of the severely injured patient and intervention for hypothermia, coagulopathy and acidosis, components of the trauma triad of death, would improve shock resuscitation [19-21]. Since cardiac arrest is an evolutive injury, it has been suggested that the optimal treatment is phase-specific and includes: the electrical phase (0–4 minutes), the circulatory phase (4–10 minutes) and the metabolic phase (beyond 10 minutes after cardiac arrest) [22]. In any case, early initiation of cardiopulmonary resuscitation is the most effective measure [23].

Inflammatory pain is caused by tissue damage [24] and its pathogeny also seems to be phase-specific. Thus, after the initial electrical phase, with upregulation of ionic channel expression in the nociceptive circuits that causes the spontaneous neural firing [24,25], the following would be an immune phase, with cytokines, chemokines and prostaglandins derived from glial and immune cells, acting as pain mediators and modulators [26,27]. Lastly, in an endocrine phase, neurotrophic factors, including nerve growth factor (NGF), brain-derived neurotrophic factor (BDNF) and neurotrophins 3 and 4, would be associated with structural neural remodeling [28]. If so, the velocity in which the phases of inflammation are expressed in the neural tissue would allow it to play a modulating role in the post-mechanical injury inflammatory response in the rest of the tissues and organs of the body.

An immediate component of the stress response to pain is the efferent nervous response mediated by the somatic motor and autonomic nervous systems [29]. The somatic motor response usually consists in the withdrawal of the affected part of the body from the source of irritation. Withdrawal reflexes are the simplest centrally organized responses to painful stimuli [30]. Furthermore, the fight-or-flight response is the behavioral response to a threat, in which the somatic motor response stands out [29]. With respect to the autonomic nervous system, both the sympathetic and parasympathetic nervous systems participate in inflammation. An early pathological motor response, where the smooth muscular fiber is prominent, particularly in the vascular system, is triggered [2,4,10]. The whey-face is one of the most visible consequences of these vasomotor responses.

The vasomotor response with vasoconstriction, which collaborates in the production of ischemia and vasodilation, cause the redistribution of the local vascular and systemic blood flow. The intensity and duration of this ischemia-reperfusion phenomenon will modify the color of the tissues and organs and will possibly determine their evolution during the subsequent inflammatory response. [2,4].

In this first phase of the inflammation, regardless whether it is local or systemic, the tone or group of dominating colors are those called cold colors, namely, blue and green, which produce sedative effects. In particular, the color blue, more or less dark, can be found after a mechanical injury, both local (ecchymosis) and systemic (cyanosis) (Figure 3).

The second-degree contusion initiates its evolution with edema and ecchymosis (Figure 1). The initial dark blue color of the ecchymotic lesion comes from the carboxyhemoglobin, which is the result of the bounding of carbon monoxide to hemoglobin. Then, the release of hemoglobin into the interstitial space is a phenomenon associated with hemolysis. Hemoglobin, released from red blood cells, is the major source of heme for bile pigment synthesis [31,32].

Heme is converted by heme-oxygenase (HO) forming biliverdin, with blue-green color, carbon monoxide and iron [32-34].

Three isoforms, HO-1, HO-2 and HO-3, are expressed in most tissues. HO-1 is an inducible enzyme, also known as heat shock protein 32, activated by oxidative stress and cytokines [34]. HO-1 has antioxidant activity related to the elimination of prooxidant heme, and to the antioxidant properties of biliverdin [34,35]. Interplay between HO-1 and nitric oxide synthase systems has recently been addressed. These systems share many common features and overlap in biological functions. Particularly, HO activity is involved in the inhibitory effect of NO on neutrophil migration to the inflammatory site [36].

HO-2 and HO-3 display a constitutive expression. HO-2 may have an essential role in the execution of self-resolving inflammatory-reparative processes [37]. HO-3 in turn, has a great structural homology with HO-2 and acts as a heme-sensing/binding protein [38]. HO-2 may also regulate the expression of HO-1 by modulating the cellular heme level [39]. Therefore, the pathophysiological mechanisms as a whole that are established in second-degree contusions due to their antioxidant, anti-inflammatory and reparative roles, would prevent the harmful evolution of the lesion towards necrosis. In essence, the effects are sedative where the expression of cold colors predominates.

Cyanosis, a word derived from the Greek term *kyanos*, is the blue coloration of the skin, and the mucosas are frequently associated with the traumatic pathology that have a systemic effect with hypoxia and hypotension [40,41]. Central cyanosis, with blueness of skin, lips and mucous membranes is always a manifestation of hypoxemia. As a result of hypoxemia an excess amount of hemoglobin is not saturated with oxygen; in currently accepted terminology this unsaturated hemoglobin is said to be reduced [42]. It is the quantity of reduced hemoglobin per deciliter of capillary blood that accounts for the bluish color of cyanosis [43] (Figure 3).

#### - Warm Colors

During the immune phase of the inflammatory response, the colors tend to be warmer. Thus, yellow coloration arises.

The bruised tissue becomes yellowish because of the emergence of bilirubin, a bile pigment [31]. Bilirrubin is produced via reduction of heme-derived biliverdin by biliverdin-reductase [31,32]. However, biliverdin-reductase, an evolutionarily conserved protein found across the spectrum of metazoans, also serves in a catabolic pathway. Homologues of the reductase are found in unicellular organisms and plants [44,45]. Plants use biliverdin produced by ferredoxin-dependent heme-oxygenase for the synthesis of phytochromes, the sensory photoreceptors [44,45].

Biliverdin-reductase may function as a protein-kinase [44]. Thus the functions are broadened since protein phosphorylation by kinases and dephosphorylation by phosphatases are essential components and mechanisms of signal transduction in the cell [44]. So, biliverdin-reductase plays an important role in mediating cytoprotective effects of HO-1 against hypoxia induced injury [44,46]. Also the existence of a link between biliverdin-reductase and the cytokine-activated stress signaling, suggest its main role in mediating the inflammatory response [44].

Bilirubin has a number of new and interesting biochemical and biological properties [47]. In addition to having a protective role against oxidative stress [47,48] bilirubin also has antiapoptotic [47,49] and antimutagenic properties [49]. Therefore, the increase in the production of bilirubin in the bruised tissue may have beneficiary effects as an inflammatory modulator.

In the immune phase of the inflammatory response, the interstitium is infiltrated first by platelets and later by leukocytes [5,50-52]. Acute inflammation following injury is the site for abundant production of ROS by phagocytic NADPH oxidase. In turn, this active oxidase is composed of a membrane-bound cytochrome [5]. In these injured tissues showing oxidative stress, and sometimes, symbiosis of the inflammatory cells and bacteria, the degree of enzymatic stress could increase [11].

Pyogenic bacteria, such as *Staphylococcus aureus*, makes the inflammatory process yellow [53]. The genus *Staphylococcus *describes a grapelike cluster of bacteria found in pus from surgical abscesses, since *staphylo *means grape in Greek. *Aureus *is the species name, and means golden in Latin, that is its characteristic surface pigmentation in comparison with less virulent S*taphylococci*. Studies of the S*taphylococcus aureus *pigment have unraveled a biosynthetic pathway that produces carotenoids, which are also a type of plant coloring with antioxidants [53]. Although this is not a tetrapyrrholic derived pigment, its situation in the scale of warm colors is interesting.

The formation of yellow, milky yellow, greenish yellow or white-yellow pus characterizes suppuration or purulent inflammation [54,55] (Figure 3). In addition to the enzymes released by granulocytes during the process of phagocytosis and bacterial killing, the bacteria themselves produce a number of exoenzymes that cause tissue destruction as well as localization of infection [56,57]. In particular, almost all *Staphylococcus aureus *strains have the ability to secrete an array of enzymes including nucleases, proteases, lipases, hyaluronidase, and collagenase [57]. Matrix metalloproteinases would also collaborate in the development of enzymatic stress in the acute inflammatory tissue injury [58,59]. Pus mainly contains necrotic tissue debris and dead neutrophils and, when the collection of pus is localized, an abscess is established [56,57].

Compensation of the acute phase response includes the production of positive acute phase proteins, like α_2_-macroglobulin, that binds proteolytic enzymes, and α_1_-antitrypsin and α_1_-antichymotrypsin, which are inhibitors of leukocyte and lysosomal proteolytic enzymes [60]. Likewise, the natural inhibitors of matrix metalloproteinases (TIMPs) could promote antienzymatic stress [58].

Also, unconjugated bilirubin is a potent inhibitor of the digestive proteases trypsin and chymotrypsin [61]. In the gut, bilirubin glucuronides are deconjugated by beta-glucuronidase, which exists in the gut mucosa, and could also be also found in some strains of bacteria such as *Escherichia coli *and *Streptococcus pyogenes*. Therefore, it has been accepted that a dramatic decrease of beta-glucuronidase-positive bacteria, which in turn results in impaired inactivation of digestive enzyme from the pancreas in the large intestine would favor the development of inflammation in this location [61,62].

The ability of S*taphylococcus aureus *to cause infection is absolutely dependent on the acquisition of iron from the host. Particularly, the most abundant iron source is in the form of the porphyrin heme [63,64]. That is why it has been suggested that the ultimate fate of exogenously acquired heme in *Staphylococcus aureus *depends on the intracellular and extracellular availability of both iron and heme. It also plays a significant role in the infectious process [64].

The yellowish coloring of the skin and mucosas is called icterus (or jaundice). This means yellowness, *ikteros *in Greek. Postoperative jaundice is associated with elevated serum bilirubin, mainly conjugated, above 3 mg per dl. Although hyperbilirubinemia seems to be multifactorial, perioperative hypotension and/or hypoxia are important pathogenic factors in the development of postoperative jaundice and multiple organ failure [65]. In patients with sepsis and multiple organ failure, a serum total bilirubin greater than 2 mg per dl is a significant factor in predicting mortality [66].

Jaundice is an important and transient clinical sign seen in most healthy newborns. They have hyperbilirubinemia but finding the cause is not often possible [67]. Nevertheless, increased concentrations of IL-1 beta in the colostrum from breast-feeding mothers whose infants had neonatal jaundice has been demonstrated. Therefore, cytokines could be involved in the pathophysiological events that can lead to neonatal jaundice [68].

However, the relation of the biliary pigments to infection is ambivalent since increasing serum levels of biliverdin and bilirubin were shown to be beneficial in the setting of inflammation [69]. Thus, in a mouse model of endotoxemia, a single-dose administration of bilirubin, in addition to its antioxidant effects, also exerts potent anti-inflammatory activity [69].

The maximum intensity of the immune response may be reached when an associated systemic infection is produced. Failure of the intestinal barrier resulting in bacterial translocation worsens the systemic inflammatory response syndrome in the polytraumatized patient, and it is an important etiological factor of sepsis and multiple organ failure [70-72].

Hypovolemic shock, severe hemorrhage or major surgery lead to priming the host and the exposure to a posterior bacterial stimulus can produce an excessive response to an otherwise low-grade inflammatory trigger [73,74]. Most likely a current definition of sepsis is too broad and encompasses heterogeneous groups of patients suffering similar but different immune syndromes that are historically grouped under the general diagnosis of sepsis [75].

Cholestatic jaundice also occurs in the setting of sepsis [76]. Liver abnormalities in sepsis include cholestasis and hyperbilirubinemia. Gram-negative infections used to be the cause of cholestasis associated with sepsis [76]. Hyperbilirubinemia develops in sepsis particularly in the setting of bacteriemia. Hyperbilirubinemia precedes positive blood cultures in one third of cases [77]. Bile pigments have apoptotic protective and proliferative effects in vitro, therefore caution should be exercised when generalising these functions or properties [49]. In addition to the possibility that bile pigments, like other porphyrins, interact with and neutralise mutagens, they may also have unique mechanistic effects that regulate cell apoptosis and carcinogenesis. The porphyrins, including biliverdin, bilirubin, protoporphyrin, hemin and clorophyllin are effective anti-mutagens. Particularly, bilirubin induces apoptosis in adenocarcinoma cell lines by disrupting the mitochondrial membrane potential and arresting the cell cycle through a prooxidant mechanism [49].

#### - Hot colors

Evidence shows that the intensity and duration of the nervous and immune phases of the inflammatory response condition the evolution of the last or endocrine phase. Thus, oxidative and enzymatic stress, both which dominate the initial phases of inflammation, according to their intensity and duration, would regulate the type of response that is produced during the final or endocrine phase. [2,4].

Platelets [78], mast cells [79], neutrophils [80,81], macrophages [82-84] and T cells [79,84] are characterized by expert functions in assisting and modulating the inflammatory response. Even today the potential role of leukocyte-derived neuropeptides and hormones in inflammation as a localized hypothalamic-pituitary-like axis has been proposed [85]. As the inflammatory response progresses, certain stop signals at appropriate checkpoints prevent further edema production and leukocyte traffic into tissues [83,86]. The pro-inflammatory mechanisms are counterbalanced by endogenous anti-inflammatory signals, that serve to temper the severity and limit the duration of the early phases, which leads to their resolution [83,86,87]. It has been proposed that regulatory T cells (Treg cells) have evolved to provide a complementary immunological arm to a physiological tissue-protecting mechanism driven by low oxygen tension (i.e. hypoxia) in inflamed tissues. The hypoxia-adenosinergic pathways migth govern the production of immunosuppressive molecules that have already been implicated in the activities of Treg cells. In this way, by virtue of acting in hypoxic and extracellular adenosine-rich tissue, T reg cells could exert their suppressive function with local downregulation of immune response, inducing "immunodormancy", and protection of tissues from continuing collateral tissue damage thus improving healing [88] (Figure 3).

However, the interstitium is considered as the battle field where inflammation develops [2,4,5] and its equivalent in tissues and organs is the stroma. At the same time, the most abundant cell type of tissue stroma is the fibroblast, an active heterogeneous population of cells [89]. Fibroblasts can modify the quality, quantity and length of the inflammatory infiltration during the induction of the inflammatory response [90]. Fibroblasts can also contribute to the resolution of inflammation by withdrawing survival signals and normalizing chemokine gradients, thereby allowing infiltrating leukocytes to undergo apoptosis or leave the tissues through the draining lymphatics [91]. Lastly, fibroblasts may also provide important positional cues for wound healing and tissue regeneration. In addition to their role of producing an extracellular matrix, they may facilitate angiogenesis by production and release of growth factors [89].

The color red is the first of the solar spectrum and is applied to the color of arterial blood, namely, when the blood contains oxyhemoglobin (HbO_2_). The reflectance spectra for human skin has a characteristic signature, due to the absorption spectrum of oxygenated hemoglobin in the blood, and provides leads about the evolution of primate color vision [92,93].

Oxyhemoglobin reaches the cells through the capillaries as a result of angiogenesis. This process, with neoformation of capillaries, would characterize the last or endocrine phase of the inflammatory response [4,11]. The relatively low solubility of oxygen combined with its rapid consumption, puts cells that are more than a hundred microns or so away from the atmosphere in the precarious position of relying on the microcirculation to maintain oxygen supply where an interruption in blood flow of only a few minutes can be disastrous [93]. Metabolically active tissues extract approximately 75% of all the oxygen from the blood as it passes from arterial input to venous output, resulting in significant intracellular gradients and intratissue heterogeneity of oxygen [93]. The oxygen dissociation curve of hemoglobin, a respiratory linked protein, has profound clinical importance applicable to numerous situations of health and disease, for example, in the neonatal period, aging, anesthesia, surgery, hemorrhage and septic shock [94,95].

Flesh color is the common color of the tissues due to its content of oxyhemoglobin. The ability to use oxygen, when it is disassociated from hemoglobin in the oxidative metabolism, is recovered when patients recover their capillary function and therefore, nutrition is mediated by them in the so-called endocrine or late phase. This type of metabolism is characterized by a large production of ATP (coupled reaction), which is used to drive multiple specialized cellular processes (energetic stress) with limited heat generation and it would determine the onset of healing [2,4,11].

Therefore, the blood cells that occupy the interstitial space in this latter phase of the inflammatory response are red blood cells [2,4]. To carry out this interstitial occupation, the red blood cells are transported by the newly formed blood capillaries [96] and, therefore, angiogenesis is considered to play the main role in this inflammatory period [2,4,10,11] (Figure 3).

The best way to finish the post-traumatic inflammatory response, both local or systemic, is with regeneration since the tissue and/or organ physiology returns to their normal state [86]. Regeneration is a process known well by the body since it is produced right afterwards and in particular by the epithelial tissues. Regeneration could be considered a good method of fighting against the energetic stress that the oxidative metabolism imposes on the epithelial cells [4,11].

Recently, lipoxins, resolvins, protectins [97-99] and vasoinhibins [100] have emerged as signaling molecules that regulate many cell functions and ample evidence emphasizes their role in the resolution of the inflammatory response [86]. Resolution is an active and tightly regulated process controlled by anti-inflammatory and pro-resolving mediators and cellular moities [86,98]. Emerging evidence now suggests that this process of resolution initiates in the first few hours after an inflammatory response begins [83]. Therefore, this process could be similar to other fermentation processes as in bread-, wine- and cheese-making. In the first case the flour is mixed with water, salt (edema, oxidative stress) and it ferments. Then it is baked in the oven to obtain bread.

Like in a cooking recipe, it is possible that the final product of the post-traumatic inflammatory response depends on how many components are used, like water, electrolytes, enzymes, pro-inflammatory cytokines, growth factors and hormones, as well as the time employed in each phase of the elaboration.

The ideal result is the resolution of tissue and organ recovery to a normal state. Mammals have retained much of the molecular machinery used by organisms such as salamanders, but their regenerative potential is only limited. In part, this seems to result from the rapid interposition of fibrotic tissue which prevents subsequent tissue regeneration [101]. However, there are other alternative solutions. By default, an impairment of wound healing and chronic hypoinflammation is produced. At the same time, by excess, the healing is produced by repair with fibrous scar or by fibroproliferative scars [51,84,101,102]. Chronic non-healing wounds generally are due to ischemia and multiple factors that contribute to their resistance to treatment [102]. Under conditions of chronic inflammatory hypoxia, chronic ischemic tissue requires adequate wound tissue oxygenation, among other factors, to improve the healing proccess [5]. The fibrous scar is secondary to excessive traumatic tissue necrosis with formation of rosy granulation tissue [51]. Lastly, prolonged inflammation in wounds contributes to the development of fibroproliferative scars, in other words, keloids and hypertrophic scars, both erithematous [103]. Free heme plays a major role in the expression of chronic inflammation. It activates neutrophil functions and delays neutrophil apoptosis. For these reasons heme is considered a pro-inflammatory molecule [104].

The fibrotic component of the wound healing response is mediated by myofibroblasts or by cells that gain a myofibroblasts-like phenotype; their activities include the abundant synthesis of fibrillar collagens [105]. In this way, the remodeling of tissues by fibrosis could be a useful solution to combat the energetic stress associated with the oxidative metabolism since the cellular content diminishes and the metabolic demand increases the extracellular component of reduced vitality.

During prolonged critical illness, lean tissue is wasted despite feeding; a problem that often persists even after the underlying disease has been resolved. In this chronic phase of the critical illness, the wasting syndrome is associated with a neuroendocrine dysfunction characterized by a hypothalamic rather than pituitary dysfunction [1,2,106]. During the evolution of the nervous and immune phases of the systemic inflammatory response, the body loses its more specialized functions and structures. In this progressive deconstruction, there is a depletion of the hydrocarbonate, lipid and protein stores, as well as multiple or successive dysfunction and posterior failure or necrosis of the specialized epithelium, i.e., the pulmonary, gastrointestinal, renal and hepatic ones [2,4,107].

However, consumption of the substrate deposits and the dysfunction or failure of the specialized epithelia of the body could also represent an accelerated process of dedifferentiation [2,4]. The hypothetical ability of the body to involute or dedifferentiate could represent a return to early stages of development. Therefore, dedifferentiation, although it means the risk of neoplastic transformation, can also be a form of effective defense mechanism against injury since it could make retracing a well-known route possible, that is, the prenatal specialization phase during the endocrine phase of the systemic inflammatory response. This last phase of the inflammatory response has the disadvantage that it develops in an extrauterine environment without the functional support of the mother with her placenta [2,4]. The elevated incidence of post-traumatic stress syndromes would thus be explained as a consequence of a frustrated recovery of homeostasis.

### Tetrapyrrole molecules in physiology and pathology

#### - Light, pigments and life

The importance of color in the surgical pathology could be attributed to the benefits for the diagnosis and treatment of diseases. However, this coloring can also have added-value related to its possible pathophysiological importance. This possibility has not yet been fully discovered, which would allow us to better understand its meaning in Nature.

Color depends on light, which is a kind of energy that the sun emits in the form of radiation [92,93]. The use of the sun's light energy by photosynthetic organisms provides the foundation for virtually all life on Earth [108].

Photosynthesis efficiently converts light energy to electrochemical energy for oxidation-reduction (redox) reactions. The direct products of oxygenic photosynthesis are carbohydrates and oxygen [108].

**Figure 4:**
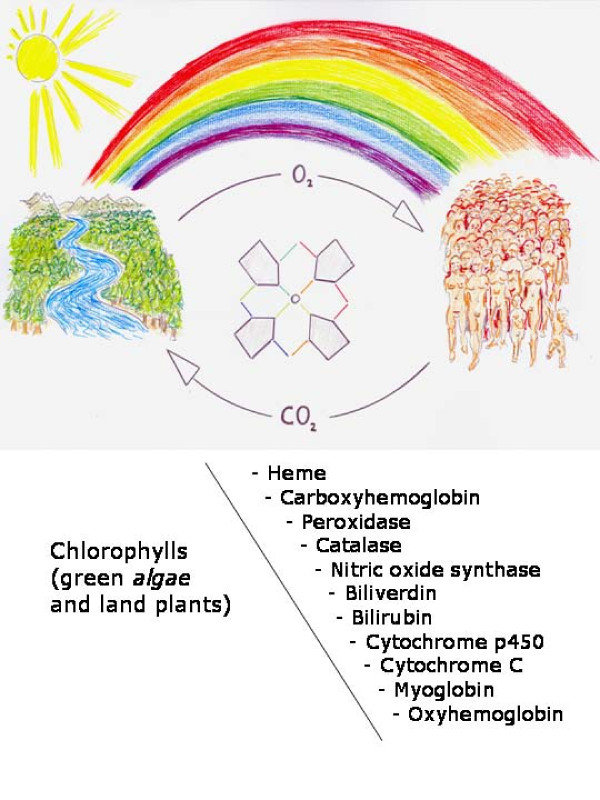
**Protagonism of the Tetrapyrrole molecules in vegetal and animal kingdoms**. Tetrapyrrole products allow plants to use CO_2_and mammals to use O_2_. These molecules in their color version take advantage of the solar spectrum, produced by the dispersion of sunlight and so they would play the main role in the origin of plant and animal life, and therefore, in inflammation.

Photosynthetic pigments are categorized in three chemical groups: chlorophylls, carotenoids and phycobilins. Chlorophylls are essential molecules of green algae and land plants. They are responsible for harvesting solar energy in photosynthetic systems but also influence processes, such as photosynthetic gene expression, growth rates and cell-death [109,110] (Figure 4).

Thus, the chlorophyll biosynthetic and degradation reactions belong to the most important biochemical pathways known [109]. However, in addition to chlorophylls, other tetrapyrrole end products are synthesized through the same pathway including heme, hemoglobin, myoglobin, cytochromes, nitric oxide synthase, peroxidase and catalases [33,109].

Tetrapyrrole molecules, such as heme, are employed in a number of biochemical processes in algae, plants [108,109], bacteria [108,111] and mammals [112] and therefore allow for establishing links between their metabolism and functions [113].

This large functional capacity of the tetrapyrrole molecules, explains why plants, through photosynthesis and mammals through respiration, are complemented in the creation of increasingly more complex forms of life [108,109,114,115]. Therefore, photosynthetic pigments and oxygen on extrasolar planets are considered strong biomarkers for detecting life [116].

#### - Pigments, oxygen and inflammation

Due to the major importance of the tetrapyrrole molecules in the evolution of life on Earth [108] we could also presuppose that these molecules play a leading role, not only in physiological situations but also in inflammation, since this is a vital process for the body.

Inflammation has been linked to the nutritional alteration in affected tissues from ancient times. In 1877 Santiago Ramón y Cajal, to obtain his doctor's degree, presented a manuscript titled *Patogeny of the Inflammation*, (the original version can be read at the Complutense University Medical School Library, although it has also been published in a facsimile edition) [117]. The future Spanish Nobel Prize winner cited the existence of disorders or perturbations of the nutritional activity in the organic territory subject to irritation, seconding Virchow. These authors considered that the essential phenomenon of the inflammatory process was irritation of the cell, which would be expressed by feeding the cell itself most actively, while exaggerating its function and by cell genesis [117].

Thus, we have proposed that the sequence in the expression of progressively more elaborated and complex nutritional systems could hypothetically be considered the essence of the inflammation, regardless of what its etiology or localization may be [2,4,5,10]. The successive pathophysiological mechanisms that develop in the interstitium of tissues when they undergo acute post-traumatic inflammation are considered increasingly complex trophic functional systems for using oxygen. The expression of the nervous (excessive oxidative and nitrosative stress), immune (enzymatic stress) and endocrine (energetic stress) functional systems during the inflammatory response makes it possible to differentiate three successive phases, which progress from ischemia, through a metabolism that is characterized by defective oxygen use (reperfusion, oxidative burst and heat hyperproduction), up to an oxidative metabolism (oxidative phosphorylation) with the correct use of oxygen that produces usable energy. Hence, the incidence of harmful influences during their evolution could involve regressing to the most primitive trophic stages, in which nutrition by diffusion (nervous phase) takes place. This is simpler, but also less costly and facilitates temporary survival until a more favorable environment makes it possible to initiate more complex nutritional methods (immune and endocrine phases) [2,4,10,11]. The ability of cells to adapt to hypoxia relies on a set of hypoxia-inducible transcription factors (HIFs) that induce a transcriptional programme of genes that regulate cell survival and apoptosis, vascular tone and angiogenesis [118]. A metabolic adaptation to hypoxia involves that cells switch from aerobic to anaerobic metabolism ("Pasteur effect"). By this mechanism the cell can continue to generate ATP and can try to meet the metabolic demands [118]. The oxygen sensors in conjunction with HIFs regulate various aspects of this metabolic adaptation [118]. Endothelial cells, through their capacity of anaerobic metabolism, could tolerate the ischemia phase and, indeed play an antioxidant role [119]

Thus, it is also tempting to speculate on whether the body reproduces the successive stages from which life passes from its origin without oxygen [120] until it develops an effective, although costly, system for the use of oxygen every time we suffer acute inflammation [4,10,11].

Oxygen availability is coupled with an increase in network complexity beyond what is reachable by any anoxic network. It also highlights enzymes and metabolic pathways that might have been important in the adaptation to the oxic atmosphere produced only by a single biological reaction: oxygenic photosynthesis. Therefore, a correlation between the increased organism complexity and the development of the use of the atmospheric oxygen could be established [120,121]. This correlation also seems to exist in the evolutive phases of the inflammatory response since progressive cellular and tissue complexity occur parallel to a gradual oxygenation process from ischemia, to progressive reoxygenation until the correct revascularization by angiogenesis in the injured tissues (Figure 4).

Tetrapyrrole end products also accompany the evolution of the inflammatory response from the beginning with ischemia to the end with oxidative phosphorylation. Thus, traumatic injury with cell damage and hemolysis can lead to high tissue concentrations of free heme, causing oxidative stress [122,123] and chemotactic call for leukocytes [122]. Catalase and peroxidase have an antioxidative effect [33]. Biliverdin and bilirubin downregulate pro-inflammation [36,47-49,69]. Hemoglobin transports oxygen in the erythrocytes and cytochrome-C-oxidase is the terminal enzyme in the respiratory chain which allows for the synthesis of ATP, where the energy of food consumption and respiration is stored [124]. The five different cytochromes in the respiratory chain constituting a family of colored proteins that are related by the presence of a bound heme molecule whose iron atom changes from the ferric to ferrous state whenever it accepts an electron. Hemes in different cytochromes have a slightly different structure and each cytochrome has a different affinity for an electron [5,33]. Therefore, it could be considered that the continuous interaction of tetrapyrrole molecules and oxygen, dominate the inflammatory response and perhaps reflect the thorough control that animal life should carry out with regards to this toxic cell potential, which is oxygen. Perhaps this is why once oxygen reaches the capillaries of the new formed tissues, whether by regeneration or by fibroplasia, the cells have to pay a very high price to obtain energy, since they overly increase their turnover (regeneration) or reduce energy to the maximum, until acquiring a tissue with the least amount of cells, and therefore, one with very little vitality (fibrosis).

#### Potential clinical applications

Sir Alan Battersby recounts that chemists and biochemists sometimes argue over coffee, each pressing the case for the greater importance of one group of natural products relative to another. Of course, this is largely for fun since living things and their chemistry are so interlocked and interdependent that (were it possible) elimination of any one family of natural products would probably bring everything crashing down [125]. This outcome is certainly so for tetrapyrroles since they are responsible "*inter alia*", for oxygen transport (haem), electron transport (cytochrome c) and most fundamentally, photosynthesis (chlorophyll) (Figure 4). Indeed, without the chlorophylls and bilins (e.g. Phycocyanin which acts as a light haverster in algae) life as we know it should not exist on this planet [125].

That is why it could be considered that tetrapyrrole molecules would be closely related to the different types of metabolisms exhibited by injured tissue during the inflammatory response. In particular, different intermediate tetrapyrroles would correspond to each post-traumatic metabolic state. Thus, through the regulation of tetrapyrrole biosynthesis genes, intermediates would be produced [125,126] in the successive phases of post-traumatic inflammation. Therefore, the assessment of color changes in tissues, attributed to the pigment characteristics of several tetrapyrroles, would possess a value for diagnosis and prognosis, and they would correlate with the metabolic level of the inflamed tissue. In essence, this correlation is also produced in the plant kingdom. Thus, the color changes that occur during foliar senescence have also demonstrated that they are directly related to the regulation of nutrient mobilization and re-absorption from leaf cells. Chlorophyll is degraded through a metabolic pathway that becomes specifically activated in leaf senescence. Furthermore, bright autumn colors observed in the foliage of some woody species have been hypothesized to act as a defense signal to potential insect herbivores [127].

A multicolor digital image analysis system for simultaneous identification of the tetrapyrrole pigments in the inflamed tissue and assessment of their metabolic activity would constitute a diagnostic method of great interest (see appendix). A rapid and simple multicolor image analysis has been developed recently for simultaneous identification of bacteria species and assessment of metabolic activity [128].

Undoubtedly, other alternatives would include experimental and clinical applications of metabolomics. Metabolomics, an *omic *science in biological systems, is the study of global metabolite profiles in a system (cell, tissue or organism) under a given set of conditions [129,130]. Metabolomics, when used as a translational research tool, can provide a link between the laboratory and clinic, particularly because metabolic and molecular imaging technologies such as position emission tomography and nuclear magnetic resonance spectroscopic imaging enable the discrimination of metabolic markers non-invasively in vivo [130]. Gas chromatography and liquid chromatography-mass spectrometry are also important analytical techniques for metabolomic analysis [129,131,132]. Therefore, the fusion of molecular/metabolic, and anatomical/morphological information could improve the diagnostic accuracy in the identification and characterization of the successive phases of the post-traumatic inflammatory response in relation to the metabolism of tetrapyrroles.

## Conclusion

We could conclude that the close relationship that the tetrapyrrole end products establish with oxygen to acquire forms of life on Earth are based on oxidative metabolism. This would also explain the tetrapyrrole end products location in the successive phases of the inflammatory response and so, phylogeny could be recapitulated [5,133] (Figure 4). Furthermore, the profusion with which nature uses tetrapyrrole derivates, including pigments in virtually all living organisms on Earth [116,134], could make possible their incorporation into our diagnostic and therapeutic arsenal. Then, the final aim of their use in the clinical area would be to achieve a similar efficiency in maintaining our life, when threatening factors arise.

## Abbreviations

ATP: Adenosin triphosphate; BDNF: Brain-derived neurotrophic factor; CO_2_: Carbon dioxide; HbO_2_: Oxyhemoglobin; HO: Heme-oxygenase; H_2_S: Hydrogen sulfide; IL-1β: Interleukin 1-beta; NGF: Nerve growth factor; RNS: Reactive nitrogen species; ROS: Reactive oxygen species; TIMPs: Tissue inhibitors metalloproteinases.

## Appendix: Tetrapyrroles and other pigment compounds involved in color production and in the inflammatory response evolution

• Haem. An alternative spelling for heme

• Heme. Heme a – C_49_H_56_O_6_N_4_Fe – Cytochrome a refers to the heme A in specific combination with membrane protein forming a portion of Cytochrome C oxidase.

      Heme b – C_34_H_32_O_4_N_4_Fe

      Heme c – C_34_H_36_O_4_N_4_S_2_Fe

• Hemoglobin (Hb). A metalloprotein (globin)

      Hemoglobin A (α_2β2_) is the most common in human adults.

• Carboxyhemoglobin – Complex of carbon monoxide and hemoglobin (COHb)

• Nitrix oxide synthase (NOS) – A eukaryotic enzyme calmodulin-containing cytochrome P450-like hemoprotein.

• Peroxidase – Can contain a heme cofactor in their active site. It is an electron donor. The optimal sustrate is hidrogen peroxide (H_2_O_2_).

• Catalase – Contains four porphyrin heme groups that allow the enzyme to react with the H_2_O_2 _to form water and oxygen.

• Porphyrin – A natural pigment containing a fundamental skeleton of four pyrrole nuclei united by methine groups.

• Photosynthetic pigments:

   - Chlorophylls – A green pigment found in most plants, *algae *and

            Cyanobacteria.

         Chlorophyll a (C_55_H_72_O_5_N_4_Mg)

   - Carotenoids – Organic pigments that naturally occur in

            chromoplasts of plants and some other

            photosynthetic organisms like algae, fungus

            and some bacteria. There are two classes:

            . xanthophylls

               and

            . carotenes – A yellow-orange-red pigments

               (tetraterpenoids)

   - Phycobilins – Light capturing molecules (chromophores)

            - blue (phycocyanobilin)

            - orange (phycourobilin) and

            - red (phycoerythrobilin)

         All of them in *cyanobacteriae*.

• Biliverdin – A green pigment formed as a by-product of heme breakdown (C_33_H_34_N_4_O_6_).

• Bilirubin – A yellow breakdown product of normal heme catabolism (C_33_H_34_N_4_O_6_)

• Bilirubin glucuronides – Bilirubin glucoronidation reaction is catalyzed by UGT (uridine diphosphate (UDP)-glucuronyl transferase).

      - Bilirubin monoglucuronide

      - Bilirubin diglucuronide

• Bile salts

   - Urobilinogen is a colorless product of bilirubin reduction (C_33_H_44_N_4_O_6_)

   - Urobilin is a yellow linear tetrapyrrole produced when urobilinogen is oxidized by intestinal bacteria. This produces a brown pigment excreted in urine (C_33_H_42_N_4_O_6_).

. Cytochromes

   - Cytochrome C oxidase. The last enzyme in the respiratory electron transport chain. The complex contains two hemes, a cytochrome a and cytochrome a_3 _and two copper centers.

   - Cytochrome P450 (CYP450). A large superfamily of hemoproteins found in all domains of life. Acts as terminal oxidase in multicomponent electron-transfer chains, called P450-containing monooxygenase systems.

. Myoglobin. A globular protein containing a heme prosthetic group. It is the primary oxygen-carrying pigment of muscle tissues and responsible for making these tissues red.

• Oxyhemoglobin. Heme group contains one iron atom that can bind one oxygen molecule through ion-induced dipole forces (HbO_2_). It is the oxygen-loaded form of hemoglobin.

## Competing interests

The authors declare that they have no competing interests.

## Authors' contributions

The three authors conceived, discussed and wrote the manuscript.
